# theBIGbam: compression and interactive exploration of large-scale sequencing alignments with circular mapping support

**DOI:** 10.1093/bioinformatics/btag459

**Published:** 2026-08-21

**Authors:** Martin Boutroux, Evan Thomas, Wai Hoe Chin, Hannes Peter

**Affiliations:** River Ecosystems Laboratory, Alpine and Polar Environmental Research Center, Ecole Polytechnique Fédérale de Lausanne, EPFL, Sion, 1951, Switzerland; Alpine and Polar Environmental Research Center, Ecole Polytechnique Fédérale de Lausanne, EPFL, Sion, 1951, Switzerland; River Ecosystems Laboratory, Alpine and Polar Environmental Research Center, Ecole Polytechnique Fédérale de Lausanne, EPFL, Sion, 1951, Switzerland; River Ecosystems Laboratory, Alpine and Polar Environmental Research Center, Ecole Polytechnique Fédérale de Lausanne, EPFL, Sion, 1951, Switzerland

## Abstract

**Summary:**

theBIGbam (github.com/bhagavadgitadu22/theBIGbam) is a genome browser and alignment viewer designed for massive metagenomic and metatranscriptomic datasets. The tool takes BAM files containing read alignments, together with genome assemblies in FASTA format or annotated genome sequences in GenBank format. Alternatively, it can start from raw FASTQ reads and generate alignments using a modified mapper that supports circular genomes, enabling seamless read mapping across genome ends. theBIGbam can compress hundreds of gigabytes of input files 10- to 100-fold into dedicated databases while retaining key per-position information, including coverage depth and recurrent mismatches, insertions, and deletions between reads and the reference. These databases can be served to a local web browser, enabling interactive exploration of any contig in any sample using DNAFeaturesViewer for genome maps and Bokeh for mapping-derived features. Contig–sample pairs available for visualization can be filtered using a range of summary metrics calculated per contig, per sample, and per contig–sample pair to guide users toward the most relevant signals. Through its interactive visualization, theBIGbam facilitates the exploration of complex datasets, while its integrated database—combining assembly features, annotated features, and mapping-derived features—provides the information needed to investigate biological hypotheses systematically. Designed to complement existing browsing tools like IGV and Anvi’o, theBIGbam is particularly suited for examining misassemblies, subpopulations, microdiversity, and contig topology in large-scale datasets.

**Availability and implementation:**

theBIGbam is an open-source Rust/Python package that can be installed from Bioconda or PyPI. The source code and documentation are available on GitHub (github.com/bhagavadgitadu22/theBIGbam).

## 1 Introduction

The advent of next-generation sequencing technologies has enabled large-scale metagenomics projects with hundreds of samples and billions of reads. These approaches have been applied in landmark initiatives such as the Human Microbiome Project (Turnbaugh *et al.* 2007), and in numerous studies targeting specific microbiomes, from soils (Santos-Medellin *et al.* 2021) to plants (Arnold *et al.* 2025), glacier-fed streams (Michoud *et al.* 2025) and freshwater lakes (Garner *et al.* 2023).

These projects typically involve assembling reads into contigs, followed by annotation and, eventually, binning into metagenome-assembled genomes (MAGs). Sequencing reads are then mapped back to the assembled contigs to estimate the abundance of each contig or bin within each sample. These mapping files, usually stored in BAM format, can represent terabytes of data and are hence often discarded once contig-level abundance is estimated to reduce storage requirements.

However, read coverage across a contig is rarely uniform and variability may reflect technical artefacts from library preparation, DNA extraction and sequencing biases (Sato *et al.* 2019), but may also reveal genuine biological features. For example, variation in coverage may indicate polymorphic regions suggesting the presence of microdiversity at the subpopulation level (Bellas *et al.* 2020) or horizontally transferred regions shared among multiple organisms (Aroney *et al.* 2025). However, such signals are currently often overlooked due to the paucity of bioinformatic tools that efficiently disseminate read mapping results.

Beyond coverage profiles, BAM files also encode alignment discrepancies. For instance, contig end positions exhibiting elevated read clippings reflect inaccurate assembly boundaries, while regions enriched in deletions suggest structural inconsistencies (Li *et al.* 2023, Trigodet *et al.* 2025). Careful inspection of alignment tracks can therefore uncover technical and biological patterns that remain hidden when relying solely on global summary metrics.

Genome browsers such as JBrowse 2 (Diesh *et al.* 2023), Integrative Genomics Viewer (IGV) (Thorvaldsdóttir *et al.* 2013), and Anvi’o (Eren *et al.* 2015) enable visualization of read alignments alongside reference genomes. IGV and JBrowse 2 provide interactive, region-level inspection, allowing users to zoom in on small genomic windows or individual genes but requiring retention of the full BAM files. Anvi’o offers contig-centric summaries, compressing alignment data into databases and organizing per-contig metrics. However, this approach aggregates base pairs into bins, thereby reducing fine-scale resolution. More importantly, while these tools are powerful for targeted exploration, they are not designed to systematically interrogate massive metagenomic datasets containing millions of contigs across hundreds of samples.

Here, we present theBIGbam, a scalable framework that summarizes, stores, and visualizes per-position mapping features across large metagenomic datasets. It enables systematic exploration of contig–sample pairs at scale, guiding users visually through their datasets to develop scientific insights. To support this exploration, the tool also provides analyses and quantitative metrics including read coverage profiling, misassembly and microdiversity characterization, and even genome topology.

A key feature of theBIGbam is its explicit support for circular genome mapping. While tools such as Anvi’o, Circlator or Circle-Map can infer potential circularity by detecting reads that partially map to both contig ends, this approach does not implement true circularized alignment (Eren *et al.* 2015, Hunt *et al.* 2015, Prada-Luengo *et al.* 2019). In contrast, theBIGbam preserves coverage continuity across genome termini by mapping reads to duplicated contig sequences, thereby preventing artificial splitting of junction-spanning reads at contig boundaries. This avoids partial read loss and enables accurate circularity assessment as well as improved abundance and completeness estimates.

## 2 Methods

theBIGbam comprises three main commands. The *mapping-per-sample* command performs read alignment using an adapted version of common mappers that support circular genomes. The *calculate* command processes alignment files together with annotated assemblies to generate a database 10 to 100-fold smaller than the input files. This database can be explored interactively in a local web browser via the *serve* command.

### 2.1 Generating theBIGbam-compatible alignments with circularity support

theBIGbam is not a read aligner: it relies on the well-established mappers minimap2 (Li 2018) and bwa-mem2 (Vasimuddin *et al.* 2019) to generate outputs compatible with theBIGbam *calculate* command and to support circular genomes. theBIGbam *calculate* command requires sorted, indexed Binary Alignment Map (BAM) files. Alignment files should include secondary and supplementary alignments, as well as MD tags for variant calling. Users can generate such BAM files with standard mappers and SAMtools (Li *et al.* 2009), or directly from FASTQ files with theBIGbam *mapping-per-sample* command, simplifying the workflow.

Circular genome support allows reads to span contig ends, which is important for assessing contig completeness and topology. Standard mappers typically split these reads into a primary and a chimeric supplementary alignment, which is often discarded by default. In the circular mode of theBIGbam *mapping-per-sample* command, each contig is duplicated prior to alignment, enabling seamless mapping across the junction. Artificial secondary and supplementary alignments arising from the duplication are removed, and reads are reassigned to their correct positions before output. This approach preserves consistent coverage at contig ends of circular genomes. For linear genomes, circular mapping has negligible effects on terminal bases. theBIGbam can be used solely as a circular genome extension to produce standardized alignments for viral genomes, plasmids, bacterial chromosomes, and organellar genomes (The SAM/BAM Format Specification Working Group 2024).

### 2.2 Extracting genomic and mapping-derived features

The *calculate* command of theBIGbam converts large BAM files and associated annotated assemblies into a compact, queryable DuckDB database (Raasveldt and Mühleisen 2019). Implementation in Rust and parallelization across samples and contigs ensure high performance. Twenty-eight features distributed across one contig module and six mapping-derived modules are computed from input files.

#### 2.2.1 Contig module

This module provides genomic context for interpreting mapping-derived features. For example, localized increases in coverage may reflect mobile elements such as transposases, whereas decreases may indicate assembly artefacts, including collapsed or misrepresented repeats. Sequences are stored along with sequence-derived properties compressed as continuous features. Repeats are identified by self-aligning each contig using BLAST with a minimum e-value of 1e^−10^ (Camacho *et al.* 2009). GC content and GC skew are computed over non-overlapping windows of 500 bp and 1 kbp, respectively. When available, gene coordinates are retained together with amino acid sequences and other annotated features (e.g. tRNAs).

#### 2.2.2 Mapping-derived modules

**Figure 1:**
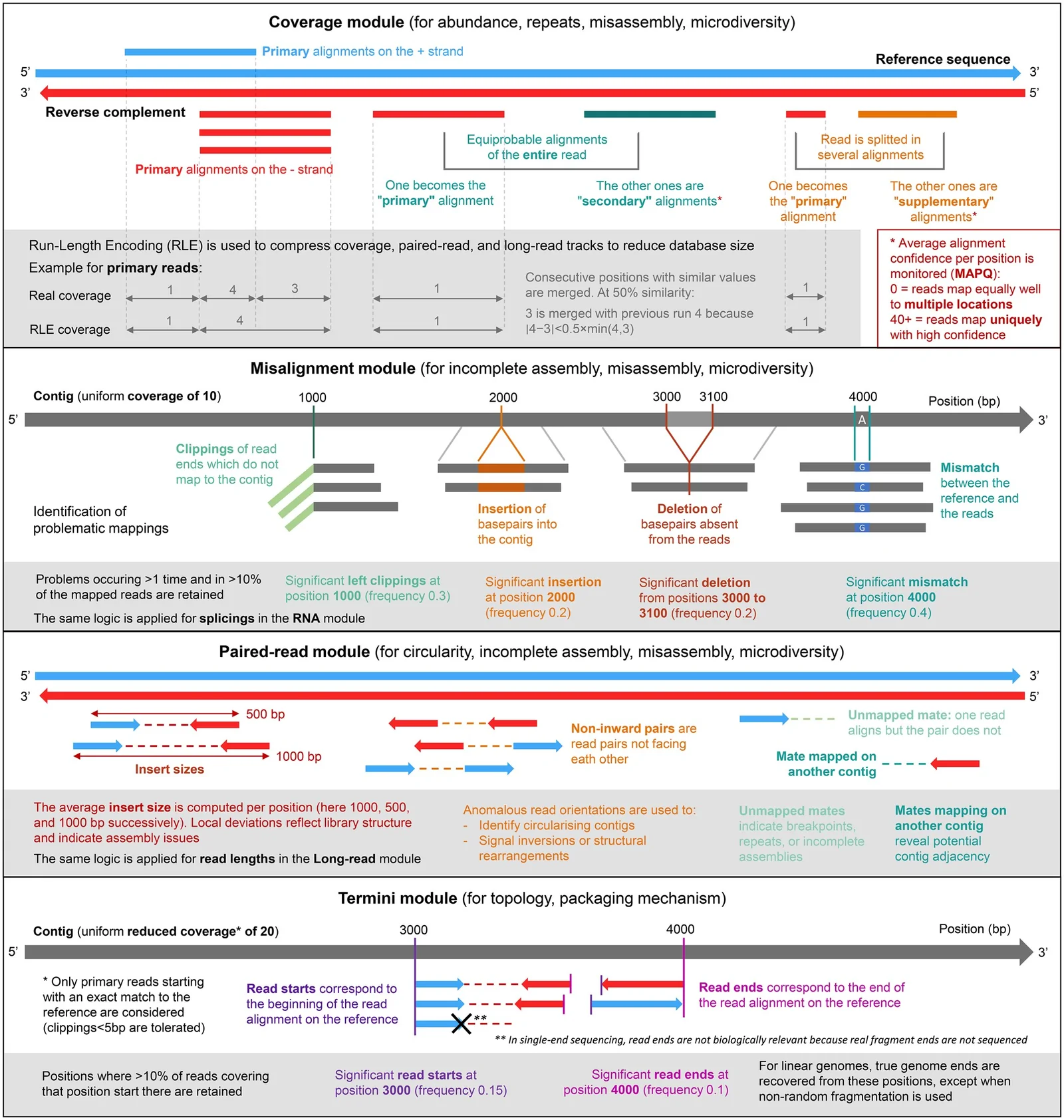
Overview of features from the six mapping-derived modules. Grey boxes contain explanation to the respective illustrations. The run-length encoding (RLE) method used for continuous features (represented as curves) is illustrated in the coverage module.

Six mapping-derived modules summarize per-position features from BAM files (Fig. 1). Each BAM file represents a single sample, with one record per read indicating the reference contig it aligns to and the alignment details. Reads mapping to each contig are processed sequentially, and per-position arrays are used to accumulate values for all mapping-derived features. To achieve a lightweight database, only contigs in samples with sufficient read support are retained (default: at least 50% aligned fraction). In addition, continuous features, such as coverage profiles, can be compressed using run-length encoding (RLE), grouping consecutive windows into a single run and creating a new entry only when values vary beyond a user-defined threshold (disabled by default). Discrete events, such as mismatches, are retained only at positions where they occur more than a user-defined minimum number of times and exceed a minimum fraction of the local coverage, thereby focusing on the most significant events (default: 2 and 10%, respectively).

The coverage module comprises six continuous features capturing per-position alignment depth of primary reads by strand and across both strands, the alignment depth of secondary and supplementary reads, and the average mapping quality (MAPQ) at each position. MAPQ scores estimate the probability that a read is correctly mapped.

The misalignment module comprises five discrete features derived from the CIGAR (Compact Idiosyncratic Gapped Alignment Report) string and the MD tag. In a BAM file, the CIGAR string and the MD tag provide concise representation of differences between an aligned read and the reference sequence, enabling base-by-base characterization of discrepancies. Bases present in the read but absent from the reference are recorded as insertions when occurring within the read, and as left or right clippings when occurring at read ends mapping towards the 5′ or 3′ side of the reference, respectively. Bases present in the reference but absent from the read are recorded as deletions, while single-nucleotide differences between the read and reference are recorded as mismatches. Positions supported by a sufficient number of reads showing the misalignment event are retained, and the dominant alternative sequence is recorded. In the case of mismatches, this further enables classification into synonymous, non-synonymous, or intergenic events, as well as prediction of the amino acid encoded by the alternative sequence. For alignments generated with RNA-aware aligners such as HISAT2 (Kim *et al.* 2019), the RNA module can also infer the positions of splicing events from the CIGAR string.

The paired-end short reads and long reads modules extract information specific to their respective sequencing types. For long reads, the lengths of reads mapping at each position can be informative; for instance, regions with consistently shorter reads may indicate secondary structures (e.g. hairpins) that cause fragmentation during DNA preparation. Average insert sizes in paired-end short reads provide comparable information and can also reveal misassemblies. The paired-end read module also records the number of reads whose mate is either unmapped or mapped to a different contig. For read pairs that both map to the same contig, their orientation is checked to ensure that each read maps to the expected strand.

The termini module was designed to identify termini of linear genomes, and particularly of compact packaged genomic elements such as many phages, which alternate between linear and circular topologies (Garneau *et al.* 2017). During DNA extraction, genuine genomic termini are preserved as fragment boundaries because they correspond to physical ends of the linear genomic element. During sequencing, this results in an enrichment of read alignments that begin or end at these positions, provided that the library was prepared without fragmentation or with random fragmentation (i.e. restriction enzyme digestions would not produce enrichment). These enriched positions are recorded in the database.

### 2.3 Visualization of extracted features through an interactive web interface

The resulting database can be interrogated directly using SQL but it can also be explored interactively using the Python package Bokeh (Jolly 2018). The *serve* command takes the database path as input and launches a local web server, providing a user-friendly interface where SQL-like queries are executed dynamically to the DuckDB backend, eliminating the need to load large volumes of data on the client side.

**Figure 2:**
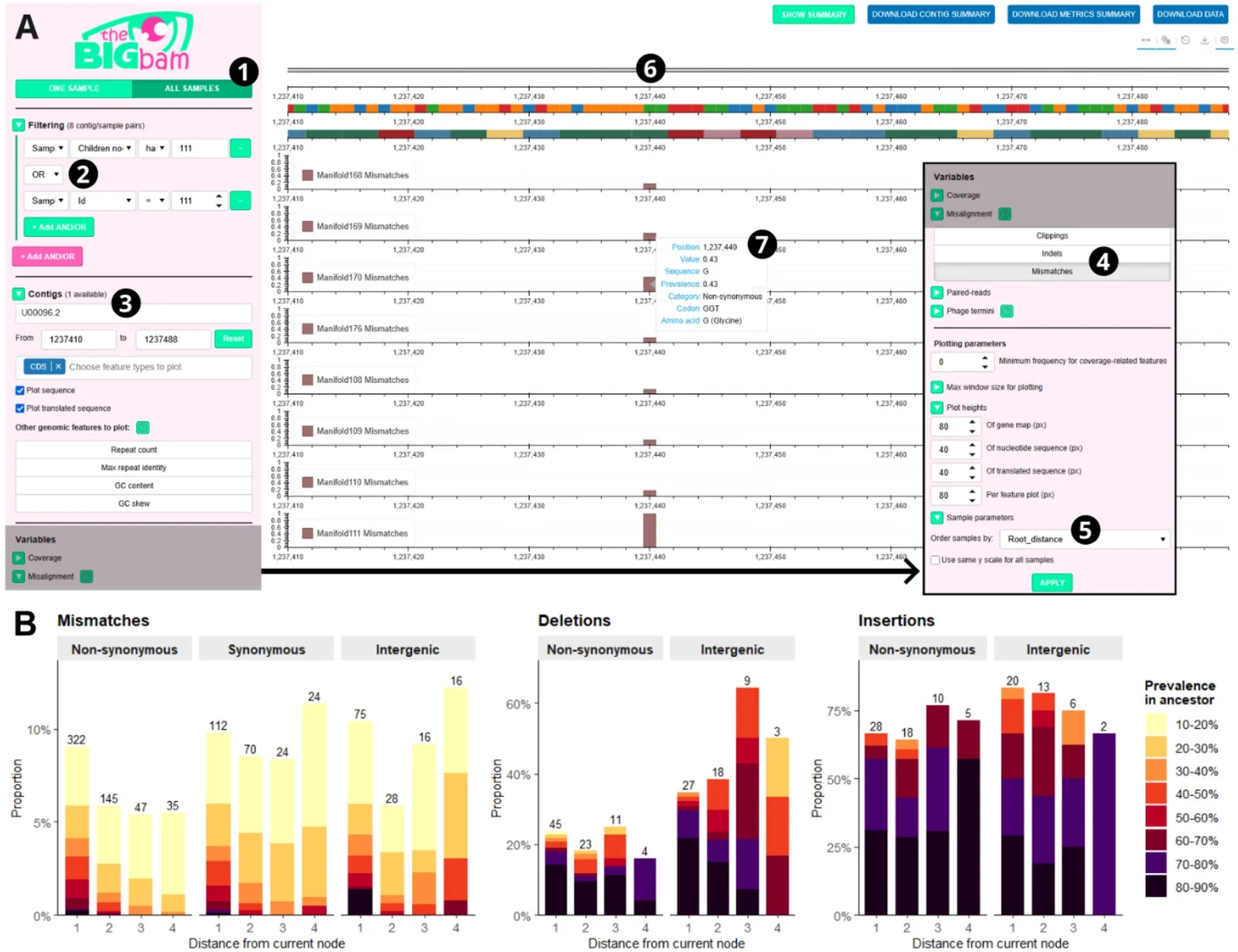
Microdiversity within the *E. coli* megaplate. (A) theBIGbam interface showing a mismatch across seven generations. **1** “All Samples” view to display the eight generations. **2** Filters applied on the contig-sample pairs available in the dataset (keeping ancestors of node 111). **3** Selection of the contig. **4** Selection of features (note arrow). **5** Ordering the plots genealogically. **6** Gene map. **7** A tooltip showing mismatch details (B) Proportions of the population carrying a mutation that later became fixed. Plots are stratified by mutation type (mismatch, insertion, deletion) and functional category (non-synonymous, synonymous, intergenic). Proportions are calculated for ancestor groups prior to the node at which the mutation became clonal. Colors indicate prevalence of the ancestral subpopulation. Numbers above each column indicate the total count of mutations already present at the given ancestral distance.

The web interface is organized into two main panels: a left panel for choosing visualization parameters and a right panel for displaying the resulting plots (Fig. 2A). The primary visualization mode, “One Sample,” allows exploration of features for a single contig–sample pair. Alternatively, an entire MAG can be visualized. Users select a contig, a sample, and one or more features to display from autocomplete lists generated from the database. The lists of available contigs and samples can be filtered using contig and sample characteristics, as well as metrics computed per contig–sample pair, enabling users to target specific signals or hypotheses. In “All Samples” mode, users can compare the profile of a mapping feature for a contig across multiple samples.

After confirming the selection (“APPLY”), the corresponding plots are rendered in the right panel. Leveraging Bokeh’s interactive capabilities, users can zoom, pan, and hover over data points to access additional metadata, as well as export the current view as a PNG image or download data as a CSV file. When an annotation file is provided, genomic features can be displayed using the DNAFeaturesViewer package (Zulkower and Rosser 2020). Underlying nucleotide and amino acid sequences of annotated proteins can also be shown. This contextual information facilitates interpretation of mapping signals within their genomic context.

### 2.4 Local and contig-wide metrics to derive computational and biological insight

The tool was designed to provide visual access to genomic and mapping-derived characteristics for any contig in any sample. However, metagenomic datasets can be extremely large, making it difficult to identify informative contig–sample pairs without specific hypotheses. To guide interpretation, summary metrics are computed for each contig–sample pair across four primary use cases: presence detection, misassembly identification, microdiversity analysis, and topology characterization. For MAG datasets, relevant metrics are also computed at the MAG level.

#### 2.4.1 Presence detection

Mapping files are commonly used to estimate contig abundance per sample. To this end, several common coverage metrics are provided: mean coverage, median coverage, and trimmed mean coverage (excluding the 5% of positions with the lowest and highest coverage). In addition, RPKM and TPM (Reads and Transcripts Per Kilobase per Million mapped reads, respectively) are provided to normalize for sequencing depth, enabling comparisons across samples (Aroney *et al.* 2025).

However, read mapping alone does not guarantee contig presence due to shared genomic regions across different contigs. True presence should yield a statistically meaningful distribution of reads along the contig. To evaluate this, the fraction of aligned positions is calculated, together with the coefficient of variation to capture broad deviations from the mean and relative coverage roughness to detect sharp, localized fluctuations.

Exploring contig–sample pairs under varying thresholds of these metrics can help define criteria for determining contig presence. These empirically informed thresholds can then be combined with formal statistical approaches, providing more accurate and robust identification of contigs across samples than relying on arbitrary cutoffs or conventions. This is important because threshold choice can strongly influence interpretation, for instance, whether contigs appear widespread or locally restricted.

#### 2.4.2 Misassemblies and microdiversity

The misalignment module flags positions where mapped reads diverge substantially from the reference. These positions may reflect subpopulations missing a segment of the contig or carrying an extra segment. Discrepancies supported by more than 50% of locally aligned reads can also be considered misassemblies when they occur in the sample from which the contig was assembled.

To quantify microdiversity and misassembly per contig per sample, base pairs present in reads but absent from the reference (collapses) and base pairs present in the reference but absent in reads (expansions) are computed at all flagged positions (microdiversity level) and at positions where > 50% of reads support the discrepancy (misassembly level) (Guo *et al.* 2025). Collapse size at each position is calculated as the average length of insertion and clipping events. The sum of collapses and expansions gives contig-level analogs of completeness and contamination.

These metrics should be interpreted as approximations rather than exact measurements, as mapping files only provide contextual information up to the read length (typically ∼150 bp for short reads). For instance, a clipping event may reflect a small (e.g. ∼200 bp) or a large (e.g. >10 kbp) missing segment, yet the inferred length is inherently capped by read length (e.g. ∼100 bp for short reads). As a result, those metrics tend to underestimate the size of missing or extra genomic segments.

#### 2.4.3 Topology characterization

Complete contigs can be either linear or circular. Circularity is supported by reads that map across both ends of a contig, which can be accurately detected when mapping is performed with theBIGbam in circular mode. With paired-end data, circularity can also be inferred—albeit with lower precision—when read pairs map to opposite ends of the contig. Both types of circularizing reads are counted and reported as complementary metrics. In the absence of such reads and clipping events near the contig termini, a linear structure is inferred.

In addition, the termini module enables the identification of termini in packaged genomes, including phages. Users can visually inspect contigs to locate putative termini and compare these signals with packaging mechanism predictions generated by theBIGbam or tools such as PhageTerm (Garneau *et al.* 2017).

## 3 Results

### 3.1 Performance of theBIGbam

Database construction and interactive exploration were tested to ensure efficient performance on large datasets in terms of runtime and computational resources. Two paired short-read Illumina datasets were run in theBIGbam *calculate*: (i) a medium-sized dataset comprising 252 sequencing runs of mutated *E. coli* (Baym *et al.* 2016), and (ii) a large dataset consisting of 192 metagenomes collected from glacier-fed streams worldwide, 50 000 viral contigs >10 kbp and 3000 bacterial MAGs (Michoud *et al.* 2025, Boutroux *et al.* unpublished). Reads were mapped using minimap2 (v. 2.30), with theBIGbam circular mode for the *E. coli* and the viral datasets. The resulting BAM files totaled 25 GB for the *E. coli* dataset, 350 GB for the viral dataset and 3 TB for the bacterial MAGs.

With default parameters, theBIGbam converted the BAM and annotation files into DuckDB databases of 2.7 GB (*E. coli*), 36 GB (viral dataset) and 527 GB (MAG dataset), corresponding to compression factors of 9x, 10x, and 5.5x, respectively (Tables S1 and S2). Allowing partial loss of per-position information using a run-length encoding threshold of 50% reduced database sizes to 1.1 GB, 26 GB, and 473 GB, respectively, corresponding to compression factors of 25x, 14x, and 6.2x. Runtime took around 10 minutes, 1 hour, and 1 day for the three datasets, respectively. This demonstrates near-linear scaling of runtime with input size and the ability of theBIGbam to efficiently process massive datasets.

Visualization performance was also assessed. Querying and rendering individual contig–sample pairs from the 36 GB viral dataset incurred minimal latency. Rendering a 30 Mbp chromosome from the *Arabidopsis thaliana* reference genome (GCF_000001735.4) also worked without noticeable lag, indicating efficient performance even for large contigs.

### 3.2 Effect of circular genome support on read mapping

Circular mapping is expected to benefit all complete circular elements, particularly smaller contigs, where terminal regions constitute a larger fraction, and long-read datasets, where reads are more likely to span contig boundaries. To illustrate this, we mapped both short and long reads to the 39.7 kbp genome of the model bacteriophage HK97 (NC_002167.1) using theBIGbam *mapping-per-sample* command in default and circular modes. Circular mapping approximately doubled coverage at both contig ends. At the 3′ end, circular mapping increased coverage from 100 to 197 for short reads and from 2482 to 6337 for long reads (Fig. S1B). The region of improved coverage spanned approximately half the average read length (e.g. 75 bp for short reads) from each contig end. Circular mapping using long reads increased the global mean coverage from 4956 to 5706.

### 3.3 Assessment of viral prevalence

Ecological analyses often rely on taxa presence/absence across samples (i.e. taxa prevalence). However, read contamination can inflate contig prevalence in metagenomic datasets, potentially leading to misleading conclusions (e.g. “everything is everywhere”). To evaluate this effect, we analyzed the prevalence of viral contigs across 192 geographically distant samples. Without any filtering threshold, 52% of viral contigs were detected in more than 90% of the samples. This result appears unlikely given the strong spatial isolation and large geographic distances between sites.

Visualization of coverage profiles suggests that this apparent cosmopolitan behavior is largely driven by inappropriate thresholding. Only eight of these contigs had an aligned fraction above 50% and a coverage trimmed mean above 10. Examination of these eight contigs revealed highly variable coverage profiles that poorly support the hypothesis that they originate from a single organism (Fig. S2). Instead, different segments of the contigs are likely shared among a group of widespread organisms with variable abundances. These organisms may maintain relatively stable abundances relative to one another, which could explain the consistent per-position coverage profiles observed across samples (Fig. S2).

### 3.4 Microdiversity within the *E. coli* experiment

To illustrate how theBIGbam can help investigate fine-scale evolutionary patterns, we reanalyzed a temporal dataset in which *E. coli* K-12 was grown on an agar megaplate with increasing concentrations of the antibiotic trimethoprim. This experimental setup enabled direct observation of mutation and selection along the expanding microbial front (Baym *et al.* 2016). A total of 252 samples were collected and sequenced, allowing reconstruction of evolutionary trajectories. Using the *add-sample-metadata* command, we augmented the database with ancestral and descendant relationships among samples (SI of Baym *et al.* 2016), enabling visualization of each sample alongside its ancestors in the “All Samples” view.

Figure 2A shows a zoomed-in view of the mismatches relative to the *E. coli* genome, with samples ordered in genealogical order from the root (sample 168) to the terminal node (sample 111). This view reveals a non-synonymous mismatch (valine to glycine) in a D-amino acid dehydrogenase at position 1 237 440. In sample 111, the mutation is fixed (100% of mapped reads), yet it was already detectable in the seven preceding ancestors at frequencies ranging from 14% to 43%. These and similar observations may reflect the presence of subpopulations prior to mutation fixation, thereby broadening potential interpretations of antibiotic resistance emergence beyond strictly clonal models.

To systematically assess microdiversity across the megaplate experiment, we extracted mismatch, insertion, and deletion events displaying a near-clonal pattern (>90% of mapped reads in any sample). Events already present at >90% frequency in any ancestor were excluded, retaining only newly fixed mutations. For each remaining event, at each ancestral distance (one to four precedent generations), we estimated the probability of prior detection, stratified by mutation type, functional category (synonymous, nonsynonymous, intergenic), and prevalence level (10%–90% decile bins), the latter allowing discrimination between mutations arising from rare versus already dominant subpopulations (Fig. 2B).

Overall, 8.0%, 26.4%, and 72.3% of ancestral samples harbored a subpopulation carrying the same mismatch, deletion, or insertion that later became fixed in descendant generations. These proportions were calculated from 11497, 530, and 141 ancestor–descendant comparisons, respectively. Although deletions and insertions were substantially less frequent than mismatches, they were more likely to be present in ancestral subpopulations prior to fixation, rather than appearing de novo. Moreover, when detected in ancestral samples, mismatches generally occurred at lower prevalence than deletions and insertions. This may reflect the greater tolerance of single-nucleotide substitutions by DNA replication and repair systems, allowing mismatches to persist at intermediate frequencies even in the absence of a substantial fitness advantage. This interpretation is further supported by variation in single-nucleotide substitutions across functional mutation categories. Across all event types, non-synonymous mutations were least likely to be detected in ancestral samples prior to fixation. This difference was significant for mismatches and deletions (Fisher’s exact test, *P* < .01), with a similar, though non-significant, trend for insertions. These results suggest that non-synonymous mutations rarely persist at intermediate frequencies unless strongly beneficial, whereas synonymous and intergenic mutations are more readily tolerated within subpopulations. Moreover, non-synonymous mismatches were significantly more likely to be detected in the direct ancestor than in more distant ancestors (Fisher’s exact test, *P* < .01), indicating that these mutations often appear as subpopulations before fixation.

These findings should be interpreted in light of the relatively low sequencing depth (33× on average). In regions with coverage below 10×, a single supporting read can meet the 10% threshold used to define event presence. While sufficient for analyses assuming largely clonal populations, this depth does not support fine-scale resolution of microdiversity and increases susceptibility to artifacts such as index hopping, which can inflate apparent variation. Although individual subpopulations carrying specific mutations cannot be confidently resolved, the recurrent presence of mutations as ancestral subpopulations across multiple evolutionary trajectories supports the conclusion that these populations were frequently microdiverse rather than strictly clonal. theBIGbam enabled visual exploration of this hypothesis and its quantitative characterization.

## 4 Discussion

In this work, we introduced theBIGbam, a tool designed to produce and compress large BAM alignment files into compact databases that retain key per-position mapping features and can be explored through an interactive web interface. theBIGbam enables scalable and intuitive exploration of large omics datasets. It reports read-alignment–supported events with minimal interpretation, while the accompanying metrics provide guidance to help users interpret the data. A limitation of this approach is the necessary tradeoff of losing individual read information to achieve a lightweight database and extract signals from noisy data. Moreover, compression can dilute smaller signals; this effect can be minimized or even eliminated by adjusting the default compression parameters if preserving exact profiles is crucial and database size is not a concern.

To our knowledge, theBIGbam circular mapping mode, which extends standard mappers, represents the first open-source implementation of explicit support for circular genomes during read alignment outside of the proprietary software CLC Assembly Cell (CLC bio 2010). The BAM files produced in this mode follow the SAM/BAM specifications for circular references. However, because practical support for circular references remains limited in widely used open-source tools, using these files outside theBIGbam may lead to unexpected behavior at contig boundaries. Another limitation concerns MAPQ scores: because the reference is duplicated, reads can align equally well to both copies, resulting in artificially low MAPQ values. Future versions of theBIGbam will aim to implement a more rigorous and internally consistent MAPQ recalculation.

We envision theBIGbam as a tool for deep exploration of alignment-rich omics datasets, which are often underutilized. Although exhaustive inspection of contigs across many samples is infeasible, targeted analysis of selected (or even randomly sampled) contig–sample pairs can yield valuable insights and help assess assembly and alignment quality. Direct visualization of alignment patterns can generate biological hypotheses that are not readily captured by aggregated metrics, as illustrated by the unexpected microdiversity observed in the megaplate dataset. theBIGbam was mainly tested on microbial metagenomic datasets but it is broadly applicable to other types of datasets, including eukaryotic genomes and metatranscriptomes. theBIGbam can be used as a one-time compression step for a complete sequencing project, integrating all contigs and reads into a single database. Alternatively, databases can be incrementally extended as new samples and contigs are generated, making the tool well suited for long-term projects such as biobanks. We recommend that theBIGbam databases be deposited in platforms like Zenodo or Figshare. While reprocessing raw reads requires substantial computational effort and expertise, a compressed, exploration-ready database allows researchers to quickly evaluate coverage and assembly quality.

In future versions, we envision online hosting of theBIGbam, adding a contig-versus-contig module, which could, for example, identify links between contigs based on shared read alignments as well as additional metrics and features guided by community feedback.

Overall, theBIGbam expands the Swiss Army knife of genome visualization and quality assessment software by enabling interactive exploration of extremely large alignment datasets. It facilitates inspection of annotation consistency, assembly integrity, subpopulation structure, and genome termini. We anticipate that theBIGbam will become a valuable resource for the genomics and metagenomics communities.

## Supplementary Material

btag459_Supplementary_Data

## Data Availability

theBIGbam is available at github.com/bhagavadgitadu22/theBIGbam, with comprehensive online documentation. It was implemented in Python and Rust, with assistance from Claude Opus 4.5. theBIGbam codebase along with the database generated for the *E. coli* evolutionary experiment are available at Zenodo (doi.org/10.5281/zenodo.18,852,589).
