# Supplementary material for: theBIGbam: compression and interactive exploration of large-scale sequencing alignments with circular mapping support
Source: Bioinformatics. 2026 Aug 21;42(Suppl 2):btag459. doi: 10.1093/bioinformatics/btag459 (PMC13501312; doi:10.1093/bioinformatics/btag459)
Supplement: Supplementary file 1 [file btag459_supplementary_data.pdf]

TheBIGbam: compression and interactive exploration of large-scale sequencing alignments with circular mapping support

Supplementary material

Martin Boutroux[1,*], Evan Thomas[2], Wai-Hoe Chin[1] and Hannes Peter[1]

[1] River Ecosystems Laboratory, Alpine and Polar Environmental Research Center, Ecole Polytechnique Fédérale de Lausanne, EPFL, 1951 Sion, Switzerland

[2] Alpine and Polar Environmental Research Center, Ecole Polytechnique Fédérale de Lausanne, EPFL, 1951 Sion, Switzerland

**Project**	Antibiotic megaplate		Vanishing glaciers			
**DOI**	10.1126/science.aag0822		10.1038/s41564-024-01874-9, partly unpublished (Boutroux et al. in prep)			
**NCBI Accession Number**	PRJNA259288		PRJNA781406, partly unpublished (Boutroux et al. in prep)			
**Number of samples**	252		192			
**Sequencing type**	paired short-reads		paired short-reads			
**Sequencing technology**	20 Illumina MiSeq, 231 Illumina HiSeq 2500		Illumina NovaSeq S4			
**Organism**	*E. coli*		viruses		bacteria (MAG level)	
**Total number of reads**	387 MB		67 billion			
**Total size of fastq files**	126 GB		4.2 TB			
**Number of contigs**	1		50,043		2,837 MAGs (1,582,129 contigs)	
**Genbank size**	6 MB		3 GB		23 GB	
**Total size of BAM files**	25 GB		350 GB		2.9 TB	
**Cluster model**	Lenovo ThinkSystem SR630 V2					
**CPU**	Intel(R) Xeon(R) Platinum 8360Y CPU @ 2.40 GHz					
**Cores per CPU**	72					
**Number of cores used**	8		16			
**RAM available**	56 GB		224 GB			
**theBIGBam options**	default	RLE* 50%	default	RLE* 50%	default	RLE* 50%
**Time spent**	10 min	11 min	1 h 28 min	1 h 10 min	24 h 07 min	22 h 55 min
**Max memory usage**	19 GB	19 GB	33 GB	27 GB	40 GB	37 GB
**Final database size**	2.2 GB	0.8 GB	36 GB	26 GB	527 Gb	473 GB
**Compression factor**	11	31	10	14	5.5	6.2

Table S1: Performance metrics from theBIGbam *calculate* operation on the *E. coli* megaplate and glacier-fed stream datasets. The table is divided into four sections: (1) project characteristics, (2) input file sizes, (3) hardware specifications, and (4) runtime and memory usage. *RLE 50%: Run-Length Encoding of 50%, corresponds to the parameter --variation_percentage 50.

<table>
<tr><th colspan="3">Organism</th><th>E. coli</th><th>viruses</th><th>bacteria (MAG level)</th></tr>
<tr><th colspan="3">theBIGBam options</th><td>default</td><td>default</td><td>default</td></tr>
<tr><th colspan="3">Total size database (MB)</th><td>2,200</td><td>36,093</td><td>527,161</td></tr>
<tr><th colspan="3">Total size features only (MB)</th><td>2,127</td><td>29,917</td><td>379,652</td></tr>
<tr><th rowspan="18">Size per feature table (MB)</th><th rowspan="11">Continuous feature</th><td>primary_reads</td><td>335</td><td>3,65</td><td>36,794</td></tr>
<tr><td>primary_reads_minus_only</td><td>255</td><td>2,667</td><td>30,271</td></tr>
<tr><td>primary_reads_plus_only</td><td>255</td><td>2,668</td><td>30,269</td></tr>
<tr><td>secondary_reads</td><td>19</td><td>1,231</td><td>35,721</td></tr>
<tr><td>supplementary_reads</td><td>1</td><td>190</td><td>7253</td></tr>
<tr><td>mapq</td><td>40</td><td>168</td><td>40,544</td></tr>
<tr><td>insert_sizes</td><td>504</td><td>4,382</td><td>20,935</td></tr>
<tr><td>mate_not_mapped</td><td>3</td><td>2,754</td><td>23,041</td></tr>
<tr><td>mate_on_another_contig</td><td>0</td><td>1,389</td><td>24,675</td></tr>
<tr><td>non_inward_pairs</td><td>328</td><td>518</td><td>46</td></tr>
<tr><td>coverage_reduced</td><td>335</td><td>3250</td><td>28,266</td></tr>
<tr><th rowspan="7">Sparse feature</th><td>deletions</td><td>0</td><td>170</td><td>2,169</td></tr>
<tr><td>insertions</td><td>0</td><td>199</td><td>2,693</td></tr>
<tr><td>mismatches</td><td>8</td><td>4,214</td><td>49,817</td></tr>
<tr><td>left_clippings</td><td>4</td><td>981</td><td>20,709</td></tr>
<tr><td>right_clippings</td><td>4</td><td>1,126</td><td>21,689</td></tr>
<tr><td>reads_ends</td><td>18</td><td>176</td><td>2,358</td></tr>
<tr><td>reads_starts</td><td>18</td><td>183</td><td>2,402</td></tr>
</table>

Table S2. Breakdown of the database size by feature table after running theBIGbam *calculate* on the *E. coli* megaplate and glacier-fed stream datasets. The breakdown is shown only for the three databases computed with default parameters.

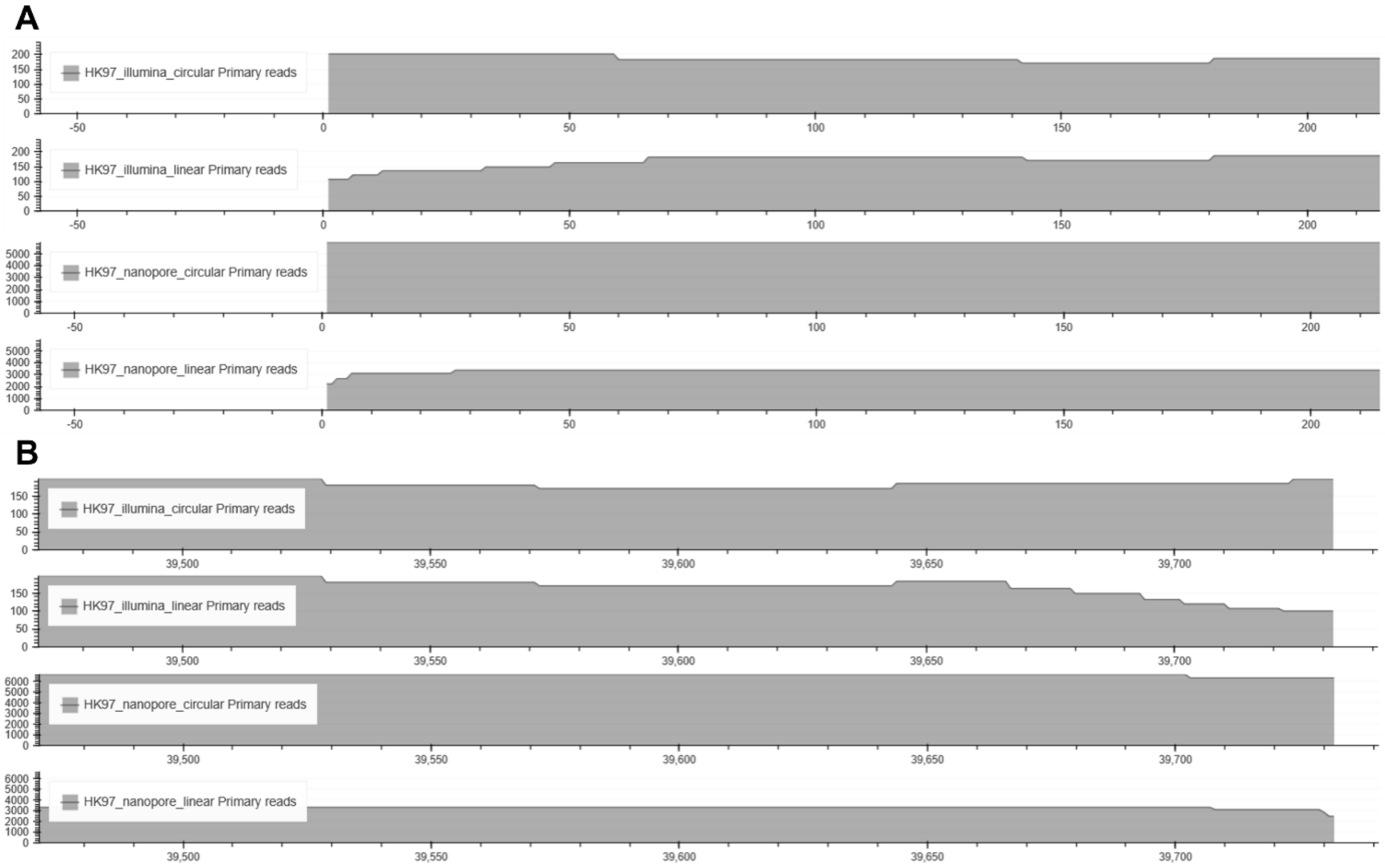

Figure S1: Coverage differences at the boundaries of phage HK97 in circular versus linear mapping modes of theBIGbam. (A) Primary read alignment depth at the 5' end. From top to bottom: Illumina short reads mapped with and without circular mode, Nanopore long reads mapped with and without circular mode. (B) Primary read alignment depth at the 3' end. From top to bottom: Illumina short reads mapped with and without circular mode, Nanopore long reads mapped with and without circular mode.

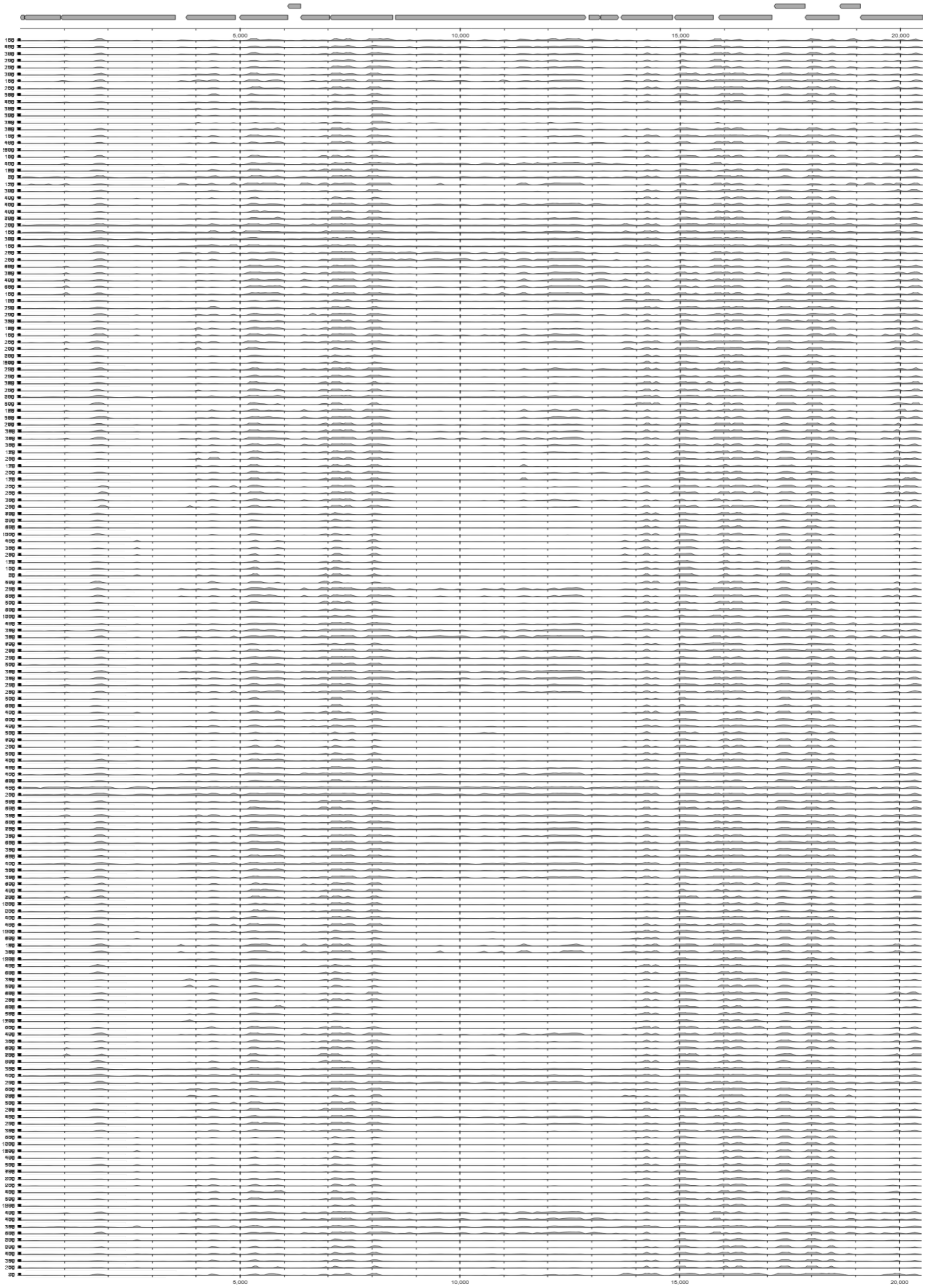

Figure S2: Gene map and coverage profiles of a cosmopolitan viral contig present in 173 samples with aligned fraction >50% and coverage trimmed mean >10. Only primary reads were included in the coverage profiles.
